# Heterogeneity in viral populations increases the rate of deleterious mutation accumulation

**DOI:** 10.1093/genetics/iyac127

**Published:** 2022-08-22

**Authors:** Brent Allman, Katia Koelle, Daniel Weissman

**Affiliations:** Graduate Program in Population Biology, Ecology, and Evolution, Emory University, Atlanta, GA 30322, USA; Department of Biology, Emory University, Atlanta, GA 30322, USA; Department of Biology, Emory University, Atlanta, GA 30322, USA; Department of Physics, Emory University, Atlanta, GA 30322, USA

**Keywords:** virus evolution, Muller’s ratchet, agent-based models, deleterious mutations, heterogeneity

## Abstract

RNA viruses have high mutation rates, with the majority of mutations being deleterious. We examine patterns of deleterious mutation accumulation over multiple rounds of viral replication, with a focus on how cellular coinfection and heterogeneity in viral output affect these patterns. Specifically, using agent-based intercellular simulations we find, in agreement with previous studies, that coinfection of cells by viruses relaxes the strength of purifying selection and thereby increases the rate of deleterious mutation accumulation. We further find that cellular heterogeneity in viral output exacerbates the rate of deleterious mutation accumulation, regardless of whether this heterogeneity in viral output is stochastic or is due to variation in the cellular multiplicity of infection. These results highlight the need to consider the unique life histories of viruses and their population structure to better understand observed patterns of viral evolution.

## Introduction

RNA viruses have high mutation rates and undergo frequent population bottlenecks, making them particularly prone to the accumulation of deleterious mutations. As such, these populations can experience deleterious mutation loads, which is the burden on fitness that recurrent and persistent mutations have on populations (Crow 1958; Agrawal and Whitlock 2012). Indeed, the accumulation of deleterious mutations in viruses has been repeatedly demonstrated using experimental evolution. In particular, experiments have demonstrated that serial population bottlenecks impact rates of deleterious mutation accumulation in viral populations (Chao 1990; Clarke *et al.* 1993; Escarmís *et al.* 1996; Elena *et al.* 1998; Poon and Chao 2004; García-Arriaza *et al.* 2005). Drugs that exploit this accumulation by increasing already high mutation rates can drive viral populations extinct (Anderson *et al.* 2004; Pauly and Lauring 2015; Bank *et al.* 2016). Experimental studies have also shown that cellular coinfection affects the rate of deleterious mutation accumulation in viral populations (Wilke and Novella 2003; Novella *et al.* 2004). In particular, cellular coinfection leads to slower purging of deleterious mutations because selection is relaxed: when multiple viral genomes are present in a cell, they all share their protein products (Zavada 1976; Froissart *et al.* 2004). With multiple copies of the same gene that have differential fitness, phenotypes and genotypes of the offspring will not necessarily be matched. Cellular coinfection therefore allows for “phenotypic hiding” of deleterious mutations (Wilke and Novella 2003; Novella *et al.* 2004).

Several processes reduce the accumulation of deleterious mutations in RNA viruses. One such mechanism is through the evolution of higher fidelity polymerase proteins, thus reducing deleterious mutation rates (Pfeiffer and Kirkegaard 2003; Coffey *et al.* 2011; Cheung *et al.* 2014). Recombination (and its segmented analogue, reassortment) also reduces the rate of deleterious mutation accumulation through the generation of high-fitness viral genotypes via viral sex. By limiting cellular multiplicity of infection (MOI), superinfection exclusion (Turner *et al.* 1999; Schaller *et al.* 2007; Folimonova 2012) also reduces the opportunity for phenotypic hiding. However, superinfection exclusion also limits the opportunity for viral sex to occur, and thus its net effect on the rate of deleterious mutation accumulation is unknown.

The effect of cellular MOI on the rate of deleterious mutation accumulation is particularly interesting to consider given its uniqueness to viral populations and that cellular coinfection is, in effect, a double-edged sword: providing an opportunity to purge deleterious mutations via viral sex, while relaxing selection on deleterious mutations by increasing the extent of phenotypic hiding. However, when phenotypic hiding dominates, the benefits of coinfection are greatly reduced for viruses that cannot recombine or reassort. Here, we develop a model to examine the effects of cellular coinfection on deleterious mutation accumulation in viral populations in the context of these opposing effects. We first show that the simplest version of the model recapitulates previous findings in the literature (Wilke and Novella 2003; Novella *et al.* 2004) that indicate cellular coinfection, in the absence of genetic exchange, increases the accumulation of deleterious mutations. We then extend this model to include cellular heterogeneity in viral output, based on experimental findings that demonstrate extreme cellular heterogeneity in response to viral infection (Russell *et al.* 2018; Martin *et al.* 2020). We find that heterogeneity, whether due to variation in cellular MOI or intrinsic cellular variation, increases the rate of deleterious mutation accumulation. Our findings highlight how viral life history characteristics can impact deleterious mutation accumulation.

## Model

### Base model

**Fig. 1.**
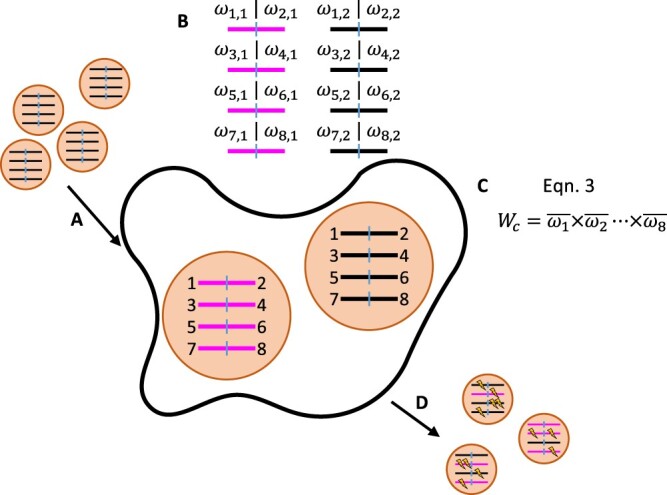
Schematic representation of the base model with a viral genome depicted over a single generation. Each generation consists of a series of steps (A)–(D). (A) *V* virions infect *C* cells. Here, 2 virions infect the shown cell. The viral genomes each have *g *=* *8 genes distributed across *y *=* *4 gene segments. Each gene is labeled 1–8. (B) Within each cell, the fitnesses of individual gene copies are calculated using Equation (1). These $\omega_{i,j}$ values are used to calculate the group fitness for each gene. (C) Cellular fitnesses are then calculated using Equation (3). (D) *V* viral progeny are formed by selecting parental cells according to their cellular fitnesses and then selecting gene segments at random from within the cell. Deleterious mutations (lightning bolts) are introduced during the formation of these viral progeny. Steps (A)–(D) are repeated for *t* generations.

We use a generalized Wright–Fisher model of the viral population (Fig. 1), with *V* virions infecting a host cell population of size *C*. Both *V* and *C* remain constant over time, yielding a constant average MOI of *V*/*C*. Each virion has *g* genes in its genome. These genes are distributed across *y* freely reassorting gene segments, with no recombination within segments. Deleterious mutations occur at a rate of *U*/*g* per gene per generation, such that the overall deleterious mutation rate occurs at a rate of *U* per genome per generation. In simulations of this model, we use y∈{1,2,4,8} to capture a range of reassortment potentials, with *y *=* *8 reflective of influenza A virus genomes. For simplicity, we use *g *=* *8 in all simulations so that genes can be evenly distributed across the considered numbers of segments. Within each gene, we adopt an infinite sites assumption. Thus, each genome can be characterized simply by how many deleterious mutations it carries at each of its *g* genes.

At the beginning of each generation, the *V* virions are randomly assigned to the *C* cells, resulting in a Poisson distribution of virions across cells. Once inside the cells, the numbers of mutations on each gene determine the aggregate fitness of the viral population within each cell. This aggregate fitness, which we call “cellular fitness,” determines the relative contribution of each cell’s virus population to the next generation of virions. To calculate cellular fitness, we first calculate the fitness of each gene that was delivered to a cell:

(1) $$\omega_{i,j}={\left(1-s\right)}^{n_{i,j}}$$

where *s* is the constant fitness cost of a deleterious mutation and $n_{i,j}$ is the number of deleterious mutations on gene *i* delivered by virion *j*. For each gene *i*, we calculate the mean fitness of the gene in a cell as

(2) $${\bar{\omega }}_{i}=\frac{1}{m}\sum_{j=1}^{m}\omega_{i,j}$$

where *m* is the MOI of the host cell. Finally, we calculate the expected cellular fitness, $W_{c}$, as:

(3) $$W_{c}=\prod_{i=1}^{g}{\bar{\omega }}_{i}$$

Equations (1)– (3) make 3 key assumptions: (1) each mutation within a gene contributes multiplicatively to the fitness of that gene [Equation (1)]; (2) each copy of a gene *i* contributes equally to ${\bar{\omega }}_{i}$ via incomplete dominance [Equation (2)]; and (3) each gene segment is essential and equally important in its contribution to cellular fitness [Equation (3)]. We make these assumptions based on the idea that when multiple virions of differing genotypes infect a cell, the produced viral proteins are treated as common goods used in the generation of progeny virions.

At the end of each generation, we draw the *V* progeny virions for the next generation from across the set of infected cells. Each progeny virion is drawn independently, with the probability that the virion comes from cell *c* proportional to $W_{c}$. Given that the virion comes from cell *c*, each of its *y* gene segments is drawn randomly from the parental virions that infected the cell. As such, a high-fitness gene segment is as likely to be drawn from a cell as a low-fitness gene segment, reflecting our assumption that cellular fitness depends on the aggregate of shared viral proteins that have been produced in a cell. Once all parental gene segments have been chosen, the mutations are added as described above. We repeat this full process for *t* discrete generations. We next describe several extensions of this base model that allow us to examine the effects of cellular heterogeneity in viral output that stem from 2 distinct sources: differences in intrinsic cellular characteristics (see *Heterogeneous Cellular Output Stemming from Differences in Cellular Characteristics*) and differences in cellular multiplicities of infection (see *Heterogeneity in Cellular Output Stemming from Differences in Cellular Multiplicity of Infection*). In *Alternative Fitness Functions*, we describe alternative cellular fitness functions that we consider a sensitivity analysis to the incomplete dominance fitness function described above.

### Heterogeneous cellular output stemming from differences in cellular characteristics

Viral output from cells can be affected by host cell characteristics such as size, cell type, and cell cycle stage (Brooke *et al.* 2013; Schulte and Andino 2014; Heldt *et al.* 2015; Golumbeanu *et al.* 2018; Leviyang and Griva 2018; Russell *et al.* 2018; Xin *et al.* 2018; Phipps *et al.* 2020; Sun *et al.* 2020). To consider the effect of heterogeneity in virus output on deleterious mutation accumulation, we extend our base model described above by adapting an approach used by Lloyd-Smith *et al.* (2005) to describe population-level viral transmission heterogeneity (superspreading dynamics). Specifically, we introduce cellular heterogeneity by making a distinction between the *cellular output* $W_{c}^{\prime }$ and the cellular fitness $W_{c}$. We make this distinction because the amount of virus produced by a cell is no longer solely determined by the cellular fitness but now also depends on stochastic factors. For each cell *c*, the cellular fitness $W_{c}$ is still determined by the genes of the infecting viruses according to Equation (3) as above. But in the next generation, the probability that a viral progeny is drawn from *c* is no longer proportional to *W_c_* and is instead proportional to $W_{c}^{\prime }$, a gamma-distributed random variable with mean $W_{c}$ and shape parameter *k*. The gamma distribution’s probability density function is given by:

(4) $$p\left(W_{c}^{\prime }=\omega |W_{c}\right)=\frac{1}{\Gamma (k)}{\left(\frac{k}{W_{c}}\right)}^{k}\omega^{k-1}e^{-k\omega /W_{c}}.$$

The parameter *k* controls the extent of cellular heterogeneity. As k→∞, heterogeneity driven by host cell characteristics becomes minimal and the probability that a progeny virion derives from cell *c* converges to its cellular fitness, $W_{c}^{\prime }\to W_{c}$. In contrast, as k→0, the probability that a viral progeny derived from cell *c* becomes increasingly dependent on host cell characteristics and relatively less dependent on the fitness of viral genes delivered to a cell. In our *Results* section, we refer to this extension of the base model as the “stochastic heterogeneity” model.

### Heterogeneity in cellular output stemming from differences in cellular MOI

Virus output from cells can also be affected by cellular MOI (Phipps *et al.* 2020; Martin *et al.* 2020). In particular, at low cellular multiplicities of infection, increases in cellular MOI can increase the average number of viral progeny from an infected cell. This increase in viral output tends to saturate at higher multiplicities of infection, indicating that at higher MOIs, there are likely constraints present on host cell machinery. In experimental studies, the specifics of the relationship between viral input and output appear to depend on the cell line and the viral strain examined (Phipps *et al.* 2020; Martin *et al.* 2020). To consider the potential for cells with higher cellular multiplicities to contribute more viral progeny than those with lower cellular multiplicities of infection, we extended the base model such that the cellular output of a cell, $W_{c}^{\prime }$, is given by the product of cellular fitness *W_c_* and the cell’s MOI *m_c_*. In the absence of viral fitness differences, this creates a linear relationship between viral input and viral output from a cell. While numerous other functional forms are possible, this is the simplest one that allows us to assess the qualitative effect of input dependence on deleterious mutation accumulation. In our *Results* section, we refer to this extension of the base model as the “input-dependent” model.

### Alternative fitness functions

To test the robustness of our results, we also consider alternative models for how cellular fitness depends on the genetic composition of the infecting virions. Above, we assume that the realized fitness of gene segment *i* is the arithmetic average of the fitnesses of the individual gene segments $i=1,\ldots ,m_{c}$. Here, we can instead consider the possibility that the fitness of gene segments depends on the fitness of the most or least-fit infecting gene segment. That is, when calculating the fitness of a gene *i*, we take either $\omega_{i}=\max\{\omega_{i,1},\ldots ,\omega_{i,m_{c}}\}$ or $\omega_{i}=\min\{\omega_{i,1},\ldots ,\omega_{i,m_{c}}\}$. These are 2 limiting models for the “dominance” of viral mutations; together with the original fitness function, they span most of the biologically plausible parameter range. We proceed to calculate *W_c_* as in Equation (3). We estimate the effects of these fitness functions under both the base model structure and with stochastic heterogeneity [Equation (4)].

## Results

In our results, we focus on presenting the mean number of deleterious mutations accumulated in a viral population by generation *t*. Unless otherwise specified, data shown are from the final generation of the simulation, *t *=* *20 or *t *=* *150. With a viral generation being approximately 5 h long for viruses such as influenza (Dou *et al.* 2017), this corresponds to approximately 4 and 31 days, respectively. We choose these 2 endpoints due to substantial changes in rates of deleterious mutation accumulation over time. Roughly, *t *=* *20 is the time to approach mutation–selection balance for many of our simulations, so changes in the number of accumulated mutations at this time reflect shifts in the mutation–selection balance distribution. At the later time *t *=* *150, we can distinguish between populations with a slow-acting Muller’s ratchet vs. ones with a fast-acting Muller’s ratchet.

### Phenotypic hiding relaxes selection

We first show that our base model reproduces key findings on deleterious mutation accumulation from previous work using similar cellular coinfection modeling frameworks, in addition to classical population genetics. That is, we establish that the sizes of the virus and host cell populations influence the rate of genetic drift and the extent of phenotypic hiding in the context of cellular coinfection.

**Fig. 2.**
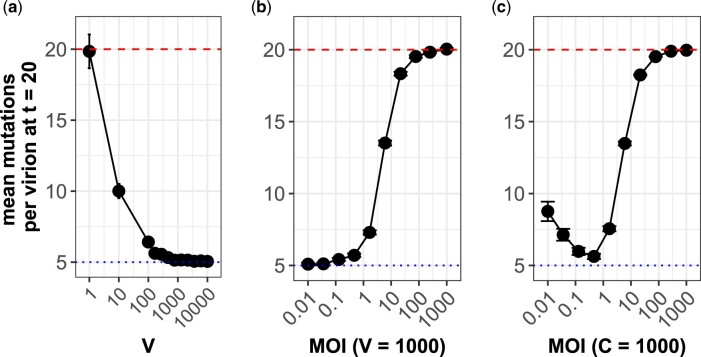
Simulated patterns of deleterious mutation accumulation without cellular heterogeneity. a–c) Mean number of accumulated mutations at *t *=* *20 generations. Each data point shows the average across 20 replicate simulations with error bars showing the standard error, except for the 3 largest population sizes in subplot (c), which have only a single replicate shown due to computational limitations. Red dashed lines show the theoretical expectation of mutation accumulation at selective neutrality (*Ut*). Blue dotted lines show the expectation of mutation accumulation for an infinite viral population size at its mutation-selection balance (*U*/*s*). Parameter values are V=1,000,C=1,000,U=1,s=0.2,g=8,y=1 unless otherwise indicated. a) Mean number of mutations accumulated across a range of viral population sizes. MOI (= *V*/*C*) is kept constant at 0.1, such that cell population sizes scale linearly with viral population sizes. Higher viral population sizes have lower rates of deleterious mutation accumulation. b) Mean number of mutations accumulated across a range of MOI. The virus population size is kept constant at *V *=* *1,000 and cell population size *C* is modified to change MOI. Here, increasing MOI increases phenotypic hiding and therefore deleterious mutation accumulation. c) Mean number of mutations accumulated across a range of MOI. The cell population size is kept constant at *C *=* *1,000 and the virus population size *V* is modified to change MOI. At low MOI, genetic drift, whose sole effects are shown in (a), dominates and mutation accumulation rates are high because of small viral population sizes. At high MOI, phenotypic hiding, whose effects are shown in (b), dominates and mutation accumulation rates are high because of high levels of cellular coinfection.

For simplicity, we begin by considering an unsegmented genome (*y *=* *1), so there is no reassortment. One key finding from the field of population genetics is that reducing population size increases the rate of deleterious mutation accumulation due to an increased rate of genetic drift, particularly in asexual populations (Fisher 1930; Wright 1931; Kimura *et al.* 1963; Lynch *et al.* 1995). This effect has mostly been studied under purely individual-level selection. This is a good approximation of our system at low MOI, where most infected cells are infected by only a single virion. Indeed simulations of our model reproduce this effect of population size at low MOI (Fig. 2a).

Previous work has also shown that cellular coinfection and the sharing of viral proteins relaxes the strength of selection on individual virions and thus allows deleterious mutations to accumulate at a faster rate in viral populations than otherwise expected (Wilke and Novella 2003; Froissart *et al.* 2004; Novella *et al.* 2004). Our model recapitulates this “phenotypic hiding” in simulations where the viral population size is kept constant and the number of cells is modified to change the overall MOI (Fig. 2b). The monotonic increase in the number of accumulated deleterious mutations in the population with an increase in MOI is directly attributable to relaxed selection.

The results shown in Fig 2, a and b indicate that increases in viral population size that are not matched by increases in the size of the cell population could yield a nonmonotonic relationship between viral population size and the rate of deleterious mutation accumulation. Indeed, Fig. 2c shows the results of this tension between the effects of genetic drift and phenotypic hiding. At low MOI (≪1), coinfection is rare, and the primary effect of increasing the viral population size across simulations is a reduction in the strength of genetic drift, thus decreasing mutation accumulation. As MOI approaches 1, however, phenotypic hiding starts to play a more pronounced role and mutation accumulation increases. At very high MOI (≫1), phenotypic hiding is essentially complete and deleterious mutations accumulate at the neutral rate *U* per unit time.

Existing analytical expressions for the rate of Muller’s ratchet provide a poor match for our simulation results, even for small MOI where phenotypic hiding is limited. For example, Gordo and Charlesworth (2000) derived analytical expressions for the rate of deleterious mutation accumulation via Muller’s ratchet in asexual haploid populations with $Ve^{-U/s}\gg 1$. Supplementary Fig. 1 shows the qualitative disagreement between their analytical equations (3a) and (3b) and our results. We attribute this to our simulations being in a parameter regime that is not often considered when modeling Muller’s ratchet, where high mutation rates are constantly introducing large-effect mutations into small populations. Approximations from Gordo and Charlesworth (2000) break down in this regime; indeed, their predictions for very small populations exceed the neutral limit of accumulation (Supplementary Fig. 1). Gordo and Charlesworth (2000) models Muller’s ratchet where $Ve^{-U/s}\gg 1$, and we only approach this regime at large *V*/*C* where phenotypic hiding is strong enough to make selection ineffective. Thus, canonical models of Muller’s ratchet do not provide a sufficient basis for prediction in the biological context of viral populations undergoing phenotypic hiding.

**Fig. 3.**
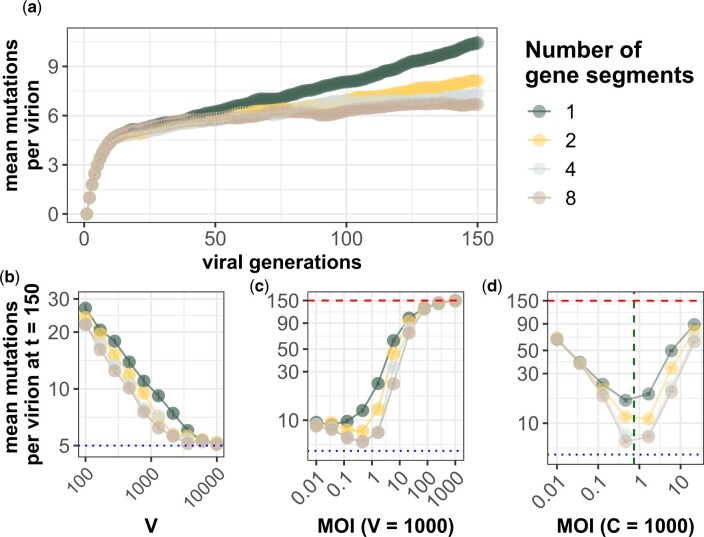
Genome segmentation slows the accumulation of deleterious mutations. In (a)–(d), the per genome mutation rate is *U *=* *1 and the fitness cost of mutations is *s *=* *0.2. Each data point is the average across 20 replicate simulations with error bars showing the standard error. Red dashed lines in (c) and (d) show the theoretical expectation of mutation accumulation at selective neutrality (*Ut*). Blue dotted lines in (b)–(d) show the expectation of mutation accumulation for an infinite viral population size at its mutation-selection balance (*U*/*s*). (a) Average number of deleterious mutations accumulated over time at a viral population size *V *=* *1,000 and a cell population size of *C *=* *10,000 for varying numbers of segments. b) Mean number of mutations accumulated across a range of viral population sizes with different numbers of gene segments. Reassortment slows mutation accumulation in small populations subject to Muller’s ratchet. MOI (= *V*/*C*) is kept constant at 0.1 by scaling linearly the cell population size *C* proportionally with the viral population size *V*. c) Mean number of mutations accumulated across a range of viral MOI. The virus population size is kept constant at *V *=* *1,000 and cell population size *C* is modified to change MOI. Even though phenotypic hiding grows stronger as MOI increases, populations with segmented genomes experience a decrease in deleterious mutation accumulation at intermediate MOI. d) Mean number of mutations accumulated across a range of MOI. The cell population size is kept constant at *C *=* *1,000 and the virus population size *V* is modified to change MOI. Segmented viral populations experience deleterious mutation accumulation when drift is strong, but benefit from reassortment as MOI increases. In both (c) and (d), mutation accumulation is slowest at intermediate MOI ≈0.3 [dashed green vertical line (d)], balancing the effects of reassortment and phenotypic hiding. At high MOI ≫1, phenotypic hiding is nearly complete and mutations accumulate at close to the neutral rate.

The rate of deleterious mutation accumulation should decrease in segmented viral genomes because reassortment can re-create high-fitness genotypes that have been lost to drift by combining segments that have a small number of deleterious mutations, halting Muller’s ratchet (Fisher 1930; Wright 1931; Kimura *et al.* 1963; Muller 1964; Haigh 1978; Chao 1990; Chao *et al.* 1997). We confirm that this occurs in our base model when we consider the viral genome of *g *=* *8 genes divided across y=1,2,4,8 gene segments (Fig. 3). Because reassortment does not affect the approach to mutation-selection balance, it has little effect at early times (e.g. *t *=* *20). At later times, reassortment results in a slower “clicking” of the ratchet (Fig. 3a), such that more highly segmented genomes end up with lower levels of accumulated deleterious mutations than genomes that have fewer gene segments. Reassortment has the largest effect on mutation accumulation at intermediate viral population sizes that are large enough to effectively select against individual mutations but small enough to be vulnerable to Muller’s ratchet, $1/s<V<e^{U/s}/s$ (Lynch *et al.* 1995; Barton and Otto 2005). At larger viral population sizes, the ratchet clicks very slowly even in nonreassorting viruses, and therefore, reassortment provides little benefit (Fig. 3b) (Muller 1964).

The higher MOI is, the more opportunities viruses have to reassort. Even when cellular coinfection and therefore reassortment is rare (MOI < 1), it can substantially slow Muller’s ratchet (Fig. 3, a–c), consistent with findings from the population genetic literature (Bell 1988; Charlesworth *et al.* 1993; Cohen *et al.* 2006). Higher reassortment rates, however, are more effective at slowing the ratchet. In Fig. 3c, we keep the viral population size (i.e. drift) the same as we test different sized cell populations to modulate MOI. As coinfection events become more common at moderate MOI, segmented genomes accumulate fewer deleterious mutations than their unsegmented counterparts. However, segmented genomes are still vulnerable to the impacts of phenotypic hiding. When cellular coinfection is frequent (MOI > 1) and phenotypic hiding causes selection to be ineffective against single mutations, mutations accumulate nearly neutrally and reassortment provides little benefit (Fig. 3, c and d, right sides). Hence, coinfection is a double-edged sword in populations with segmented genomes because sex and phenotypic hiding change the effective magnitude of selection in opposing directions. The opposing effects create an optimum MOI somewhat less than 1, at which reassortment is frequent but phenotypic hiding only mildly reduces the effectiveness of selection.

### Stochastic heterogeneity increases deleterious mutation accumulation

**Fig. 4.**
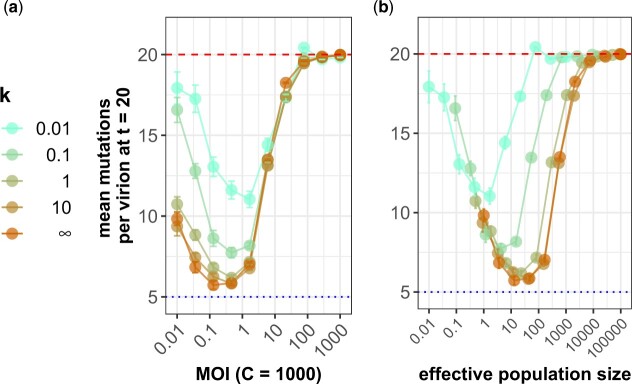
Stochastic heterogeneity increases deleterious mutation accumulation. Stochastic heterogeneity is parameterized by *k*, with k≪1 corresponding to strong heterogeneity and k→∞ corresponding to the base model without heterogeneity. a) Mean number of deleterious mutations accumulated after *t *=* *20 generations across a range of MOI, for different levels of stochastic heterogeneity. Here, the number of cells is kept constant at *C *=* *1,000, while the number of virions is increased to increase MOI. Stochastic heterogeneity has the largest effect at low MOI. At high MOI phenotypic hiding makes selection ineffective even in the absence of heterogeneity. b) Mean number of deleterious mutations accumulated by *t *=* *20 generations across simulations according to their predicted effective viral population size $V_{e}=V/(1+1/k)$. The collapse of the different curves on the left side of the plot shows that *V_e_* accurately captures the effect of heterogeneity on mutation accumulation in the regime where stochasticity is strong—small populations. In both panels, each data point shown is the average of 20 replicate simulations with error bars showing the standard error (with the exception of the 3 largest population sizes in which show only a single simulation). Red dashed lines show the theoretical expected mutation accumulation at selective neutrality (*Ut*). Blue dotted lines show the average number of mutations for an infinite viral population size at its mutation-selection balance (*U*/*s*). Parameters are C=1,000,U=1,s=0.2,y=1.

As described in *Heterogeneous Cellular Output Stemming from Differences in Cellular Characteristics*, we implement the effect of heterogeneity driven by host cell characteristics by combining individual cell heterogeneity with virus-driven differences in cellular fitness using draws from a gamma distribution, parameterized with dispersion parameter *k*. As expected, simulations with k≳1 behave like the ones described in *Phenotypic Hiding Relaxes Selection* that do not incorporate stochastic heterogeneity (Fig. 4). However, for k≪1, more deleterious mutations accumulate, with higher levels of stochastic heterogeneity resulting in faster deleterious mutation accumulation (Fig. 4a and Supplementary Fig. 2). This is because the increased stochasticity reduces the efficacy of purifying selection. This has little effect under very high mean MOI ≫1, because phenotypic hiding already weakens selection such that mutations accumulate at nearly the neutral rate, but it can greatly increase mutation accumulation at lower MOI where selection would otherwise be strong enough to halt mutation accumulation.

#### Calculations of viral effective population size show that the impacts of stochastic heterogeneity do not impact mutation accumulation at high MOI

The effect of stochastic cellular heterogeneity on mutation accumulation can be better understood by quantifying the effective viral population size, *V_e_* in these simulations. Stochastic heterogeneity in cellular virus production increases the variance in offspring number among virions and thereby decreases the viral effective population size, given by

(5) $$V_{e}\equiv V/\sigma^{2}$$

where $\sigma^{2}$ is more generally the variance in the offspring distribution (Ewens 1982). We can calculate $\sigma^{2}$ at low MOI (≪1) where almost all cells are infected with either 0 or 1 virion. Assuming that the viral population is large and ignoring fitness differences between virions, the offspring distribution is a gamma-Poisson mixture (i.e. a negative binomial) with a mean of 1 (because *V* is constant across generations) and variance $\sigma^{2}=1+1/k$. The variance expression stems from each infected cell producing a gamma-distributed number of virions infecting an approximately Poisson-distributed number of cells in the next generation. When the number of virions is small, this somewhat overestimates the variance and thereby underestimates *V_e_*, because the Poisson approximation to the binomial offspring distribution allows 1 individual to have more offspring than there are total virions in the population. Even when the number of virions is large, our formula also overestimates the variance if MOI is large, because the noise in cellular output is shared among coinfecting virions. However, this effect only becomes appreciable when the number of cells is very small.

At low MOI, we see that our calculated viral effective population size *V_e_* is indeed the relevant predictor for evolution (Fig. 4, a and b): viral populations with very different census sizes *V* but equal effective sizes *V_e_* accumulate mutations at the same rate. At high MOI, however, census size is the better quantity for evolution (Fig. 4, a and b). This is because the primary factor reducing the effectiveness of selection is phenotypic hiding, which depends on the census size (through MOI) rather than the amount of stochasticity in reproduction. Note that for the smallest simulated population sizes and *k* values we still see that our approximate formula for *V_e_* collapses the different curves, even though our approximations are breaking down and the formula is giving biologically unrealistic values of $V_{e}<1$.

### Input dependency in viral production results in slightly more deleterious mutation accumulation at intermediate MOI

**Fig. 5.**
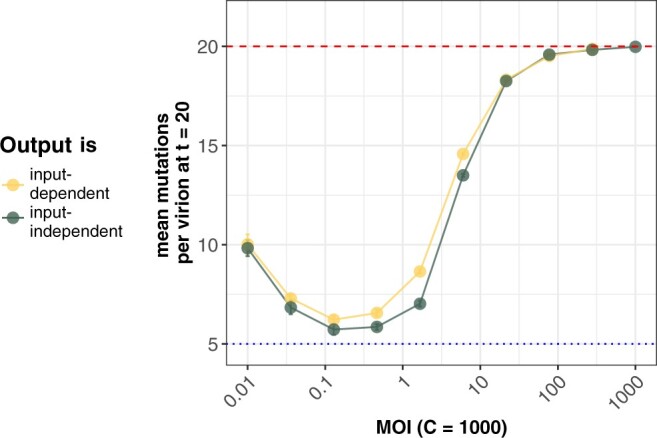
Mean number of deleterious mutations accumulated across a range of MOIs. Here, the number of cells is kept constant at *C *=* *1,000 and the number of virions is increased to increase MOI. Input dependency increases the rate of deleterious mutation accumulation only at intermediate MOIs, and only slightly at those MOIs. Parameters are *U *=* *1, *s *=* *0.2, and *y *=* *1. Each data point shown is the average across 20 replicate simulations with error bars showing the standard error. Red dashed lines show the theoretical expectation of mutation accumulation at selective neutrality (*Ut*). Blue dotted lines show the expectation of mutation accumulation for an infinite viral population size at its mutation-selection balance (*U*/*s*).

We next performed simulations under cellular heterogeneity that stems from differences in viral input. At both high and low MOIs, there was no appreciable difference between these simulations and those observed of the base model (Fig. 5). In contrast, at intermediate MOI we found slightly more mutations accumulated by *t *=* *20 generations in these simulations compared to those of the base model (Fig. 5 and Supplementary Fig. 3). To understand these results, note that at low MOI (≪1), almost all infected cells are infected by only a single virion, so the input–output relationship is irrelevant (Supplementary Fig. 3a). At very high MOI (≫1), phenotypic hiding is nearly complete and mutations accumulate near the neutral rate in both models (Supplementary Fig. 3b). At intermediate MOI, however, there is a mix of singly infected cells, where virions do not experience phenotypic hiding, and multiply infected cells, where virions experience phenotypic hiding. With viral output scaling linearly with viral input, the multiply infected cells contribute more viral progeny to the next generation, thereby increasing the representation of viral genomes that have experienced relaxed selection.

### Relaxed selection under phenotypic hiding is robust to the form of the fitness function

Above, we assume that the cellular fitness at gene *i* is determined by the average fitness of the infecting virions at the gene [Equation (2)]; here, we consider alternative models. If we think of the virions infecting a cell as being analogous to the homologous chromosomes of a polyploid individual, our model above assumes that there is no “dominance.” Here, we consider the 2 limiting possibilities of completely “recessive” or completely “dominant” deleterious mutations, in which the overall fitness of a gene is equal to the fitness of the fittest or least-fit infecting copy of the gene, respectively.

**Fig. 6.**
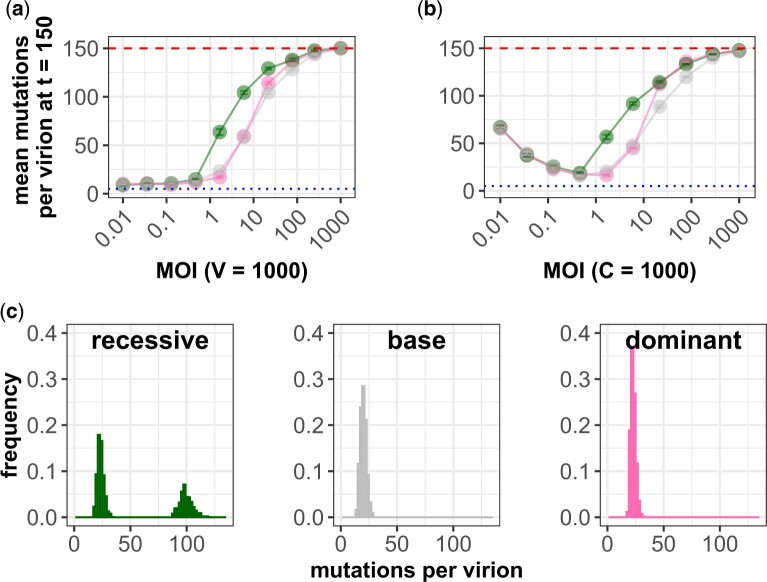
Patterns of deleterious mutation accumulation under different cellular fitness functions. a) Mean number of deleterious mutations accumulated across a range of MOIs, for different fitness functions considered. Here, the number of virions is kept constant at *V *=* *1,000 and the number of cells is decreased to increase MOI. b) Mean number of deleterious mutations accumulated across a range of MOIs, for different fitness functions considered. Here, the number of cells is kept constant at *C *=* *1,000 and the number of virions is increased to increase MOI. In (a) and (b), gray lines show results from the base model. Green lines show results from the model implementing the recessive mutation fitness function. Pink lines show results from the model implementing the dominant mutation fitness function. Each data point in (a) and (b) is the average across 20 replicate simulations with error bars showing the standard error. Parameters are *U *=* *1 and *s *=* *0.2. Red dashed lines show the theoretical expectation of mutation accumulation at selective neutrality (*Ut*). Blue dotted lines show the expected number of mutations for an infinite viral population size at its mutation–selection balance (*U*/*s*). c) Distributions of numbers of mutations per virion from a single time point of a simulation with *V *=* *1,668 and *C *=* *1,000 for each model. The recessive mutations have a bimodal distribution, with individuals tending to either have a low load or a very high load.

Our qualitative results on the effects of phenotypic hiding are robust to the form of the fitness function (Fig. 6). At low MOI, coinfection is rare, so the alternative fitness functions necessarily produce results that are essentially identical to the base case (Fig. 6, a and b, MOI ≪1; Supplementary Figs. 4 and 5). At very high MOI, simulations assuming either recessive or dominant mutations both undergo phenotypic hiding and accumulate at nearly the neutral rate, as in the base case (Fig. 6, a and b, MOI ≫1). At intermediate MOI, the primary quantitative difference appears to be that the reduced selection on recessive mutations allows them to accumulate more rapidly (Fig. 6, a and b, MOI ≈1). Interestingly, selection against dominant mutations is also less effective than in the base case for somewhat large MOI ≈30. The different fitness functions also do not change the qualitative effect of stochastic heterogeneity in increasing mutation accumulation (Supplementary Figs. 4 and 5).

While the qualitative patterns of mutation accumulation are unchanged, the fitness function can have a large effect on the distribution of mutations within the population. For recessive mutations, the distribution of the number of mutations across virions is more prone to transient bimodality, with 1 cluster of high-fitness virions and another of low-fitness ones that rely on coinfection to reproduce themselves (see Fig. 6c for an example). We think that transient bimodality occurs because phenotypic hiding allows highly loaded individuals to cheat and occasionally rise to high frequencies. Simulations where deleterious mutations are recessive are more likely to allow this phenomenon because the least-fit individuals are hidden from selective forces when they coinfect with individuals near the most-fit peak of the distribution of mutations. However, these dynamics are not the focus of the present work, as more investigation of these bimodal events requires a deeper analysis at longer time scales across a range of MOI.

## Discussion

Here, we consider how cellular coinfection in simulated viral populations impacts deleterious mutation accumulation using an in silico simulation model. Using our model, we were able to recapitulate previous results of relaxed selection under regimes of phenotypic hiding (Wilke and Novella 2003; Froissart *et al.* 2004; Novella *et al.* 2004). We then extended these findings by showing that heterogeneities experienced by viral populations, including cellular heterogeneity and differences in production of virions due to variation in number of infecting viral particles, can increase the rates of deleterious mutation accumulation.

Reassortment reduces the rate of deleterious mutation accumulation by allowing the re-creation of high-fitness viral genotypes through assembly of gene segments that each contain only a small number of deleterious mutations (Turner 2003). However, our simulations indicate that phenotypic hiding can drastically reduce this reassortment-derived benefit of segmented genomes (Fig. 3c). We show that intermediate levels of coinfection (MOI ≈0.3) are optimal for segmented viral populations since they allow sex to occur frequently enough to reduce mutation accumulation without significant levels of phenotypic hiding. While we focused on a genetic architecture based on influenza virus and therefore did not incorporate recombination into our model, we expect that recombination would give the same qualitative results as those we report for reassortment. Our parameterization of mutation rate (*U *=* *1 per genome per viral generation) was also loosely based on influenza virus (Pauly *et al.* 2017). Double-stranded and DNA viruses tend to have lower mutation rates than single-stranded RNA viruses such as influenza viruses (Peck and Lauring 2018), such that overall we expect deleterious mutations to accumulate less rapidly in populations of these viruses.

The 2 primary findings from our simulation study are that deleterious mutations accumulate rapidly in viral populations at high MOI when phenotypic hiding relaxes the efficacy of purifying selection and that fitness-independent heterogeneities in viral output from infected cells exacerbates rates of deleterious mutation accumulation. Both of these predictions can be tested through experimentation. To mirror our simulation design, these experiments would ideally maintain constant viral population sizes between generations. This can be done, for example, in serial transfer experiments by implementing single-cycle replication conditions within each dish and transferring the same amount of infectious virus at each sequential transfer (e.g. Chao *et al.* 1992; Clarke *et al.* 1993). Quantifying deleterious mutation accumulation following a given number of transfers can be done using plaque assays. Deep sequencing of viral populations can also be used to quantity deleterious mutation accumulation under the assumption that all nonsynonymous variation observed is deleterious. To explicitly test our prediction that stochastic heterogeneity increases the rate of deleterious mutation accumulation, cell lines differing in their permissiveness to infection could potentially be combined to increase the extent of cellular heterogeneity. To explicitly test our prediction that input dependency increases the rate of deleterious mutation accumulation at intermediate MOI (but not at low or high MOI), different virus strains could potentially be used, or cell lines modified to carry viral genes that would reduce the existing extent of input dependency (Phipps *et al.* 2020; Martin *et al.* 2020). While we used a positive linear relationship in our extension of the base model, exponential, saturating, and negative linear relationships may be observed. Experimentalists would need to make this consideration if direct comparisons of deleterious mutation accumulation will be made between our model predictions and empirical results.

To better understand the effects of phenotypic hiding and heterogeneities on patterns of deleterious mutation accumulation in viral populations, we have kept our simulation models highly simplified. While facilitating understanding, these simplifications make our simulations less like those of natural viral infections. Of particular note are 2 key features of natural infections that we do not incorporate into our models but that likely impact viral evolutionary dynamics. The first is that many viral infections (particularly respiratory ones) exhibit within-host spatial structure (reviewed in Gallagher *et al.* 2018). This could result in high MOI hotspots, increasing the potential for both phenotypic hiding and, in segmented viruses, reassortment. Other regions of infection could instead maintain lower MOIs, allowing purifying selection to occur more readily as long as viral populations in those regions are sufficiently large. Beyond impacting MOI, spatial structure will create correlations in viral fitness across space. This is because viral progeny are more likely to infect nearby cells, resulting in a patchy mosaic of viral fitnesses across space. The result of this patchy mosaic means that virions with similar numbers of deleterious mutations are more likely to coinfect a cell. This would reduce the extent to which phenotypic hiding can relax selection on a viral population. It would also reduce the extent to which reassortment could re-create high-fitness viral genotypes. The extent to which spatial structure in natural infections will dull the predicted impacts of cellular coinfection that we put forward here will depend on the extent of spatial structure present in the natural infection, as well as other factors such as cellular MOI that depend on viral replication dynamics.

The second key feature of natural infections that we do not incorporate into our models but that likely impacts viral evolutionary dynamics is the dramatic change in viral population sizes over the course of a natural acute infection. While our model assumes a constant viral population size, viral populations in natural infections often expand from a small founding population, reach population sizes in the millions, and then decline again until the host immune response clears the viral infection. Population expansion is known to increase the number of segregating deleterious mutations in a population but also decreases the per-individual number of deleterious mutations (Gazave *et al.* 2013). While we have not implemented simulations with variable viral population sizes between generations, we can use our existing findings to anticipate the effects these changes in viral population sizes would have on the accumulation of deleterious mutations. Based on findings from our base model (Fig. 2c), if a viral population was to grow in a limited cell population, it would experience 3 stages. First, a small founding population size would initially result in a low MOI, such that selection would be at the individual-level and deleterious mutations that would accumulate would do so as a result of genetic drift rather than phenotypic hiding. Then, selection would become more effective as the viral population expanded up until intermediate MOI. Finally, at large population sizes, cellular MOI would be high and the population would thus experience high levels of phenotypic hiding, thus relaxing purifying selection. As the viral population declined, depending on target cell availability, high MOIs might still be retained, maintaining phenotypic hiding as the dominant driver of deleterious mutation accumulation. These predictions, however, are impacted by the extent of spatial structure and patterns of target cell availability throughout infection. For example, if a viral population was to continue to colonize new tissues as it grew, MOIs could remain roughly constant, such that patterns and drivers of deleterious mutation accumulation may not appreciably change throughout an infection.

Beyond our models’ simplifying assumptions of a constant viral population size over generations and free mixing of virions, our models also assume that the fitness effects of deleterious mutations are independent of one another. One possible genetic extension of our model would be to include epistasis among mutations. Positive epistasis would result in additional mutations accumulating because the fitness effect of adding a new mutation decreases with each subsequent mutation. Negative epistasis would have the opposite effect: selection would be more strict and thus fewer mutations would accumulate. However, neither form of epistasis should have much effect on mutation accumulation at high MOI where phenotypic hiding renders mutations effectively neutral.

Phenotypic hiding can be seen as an example of social interactions between viruses at the intracellular level. The emerging field of “sociovirology” examines how such interactions between viruses, including during cellular coinfection, can have an impact on the evolution of viral populations (Vignuzzi *et al.* 2006; Andino and Domingo 2015; Bordería *et al.* 2015; Díaz-Muñoz *et al.* 2017; Sanjuán 2017; Aguilera and Pfeiffer 2019). The importance of coinfection in viral evolution has been demonstrated empirically (Chao *et al.* 1997; Turner *et al.* 1999; Wilke and Novella 2003; Froissart *et al.* 2004). Specifically, cellular MOI depends on viral traits such as aggregation via collective infectious units (reviewed in Sanjuán 2017), while other factors such as superinfection exclusion limit coinfection (Sun and Brooke 2018). Some of the other modern work in the field also highlights the role of heterogeneity (Andreu-Moreno and Sanjuán 2018; Sun and Brooke 2018). However, while much of sociovirology focuses on positive selection, our work shows that interactions among virions also have large effects on the ability of purifying selection to shape the evolution of viral populations.

## Supplementary Material

iyac127_Supplementary_Data

## Data Availability

The code used to produce the data underlying this article was written and implemented in MATLAB R2020a and is available on GitHub at https://github.com/allmanbrent/coinfection_heterogeneity. Visualization was performed using R version 4.0.1. Supplemental material is available at *GENETICS* online.
