# Supplementary material for: Heterogeneity in viral populations increases the rate of deleterious mutation accumulation
Source: Genetics. 2022 Aug 22;222(2):iyac127. doi: 10.1093/genetics/iyac127 (PMC9526070; doi:10.1093/genetics/iyac127)
Supplement: iyac127_Supplementary_Data [file iyac127_supplementary_data.pdf]

Supplementary Materials to the Manuscript: Heterogeneity in viral populations increases the rate of deleterious mutation accumulation

Brent Allman[*] [1], Katia Koelle[†], Daniel Weissman[†] [‡]

[*]Graduate Program in Population Biology, Ecology, and Evolution, Emory University, Atlanta, Georgia 30322

[†]Department of Biology, Emory University, Atlanta, Georgia 30322

[‡]Department of Physics, Emory University, Atlanta, Georgia 30322

[1]Corresponding author: Brent Allman. Emory University, Department of Biology, O. Wayne Rollins Research Center, 1510 Clifton Road NE, Atlanta, GA 30322. Phone: (619) 609-4925. Email: allmanbrent@gmail.com

1 Base Model Comparison Against Gordo and Charlesworth (2000)

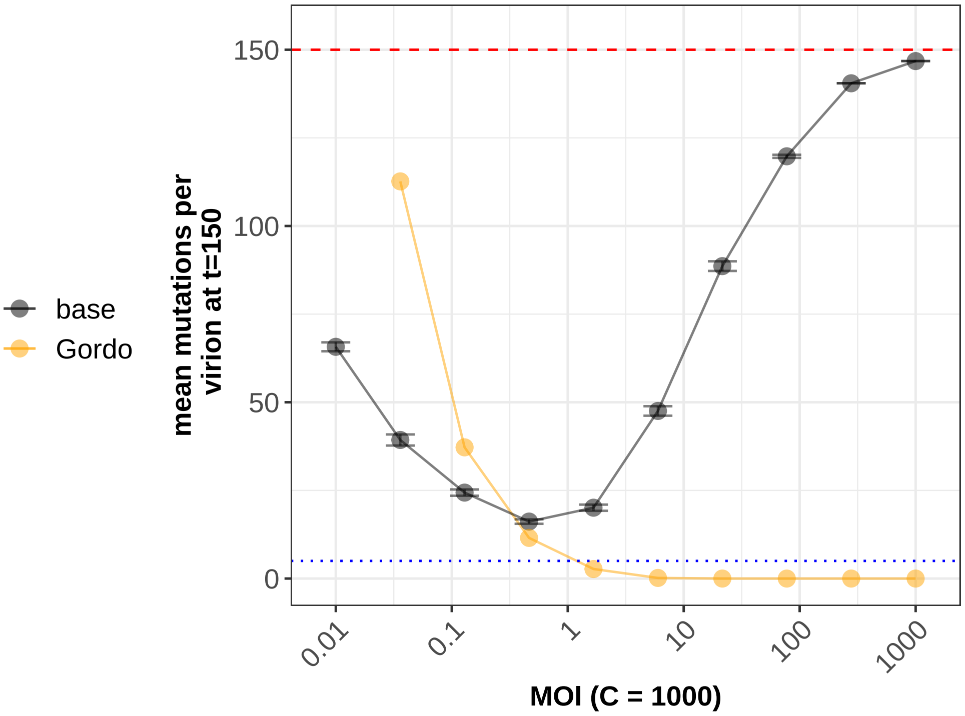

Figure S1: Mean number of accumulated deleterious mutations across a range of MOI, as simulated by our base model (black) and as analytically predicated by Gordo and Charlesworth (2000) (orange), under equivalent viral population sizes. Each data point in black is the average of 20 replicate simulations with error bars showing the standard error. Red dashed lines show the theoretical expected mutation accumulation at selective neutrality (Ut). Blue dotted lines show the average number of mutations for an infinite viral population size at its mutation-selection balance (U/s). Parameters are $U = 1, s = 0.2, y = 1$. The cell population size is kept constant at $C = 1000$ and the virus population size V is modified to change MOI. The results from our base model are not well-predicted by existing analytical results. The Gordo and Charlesworth (2000) prediction where $V = 10$ is not shown because their result far exceeds the neutral limit of mutation accumulation, likely because their model was implemented for $Ve^{-U/s} >> 1$.

2 Stochastic Heterogeneity

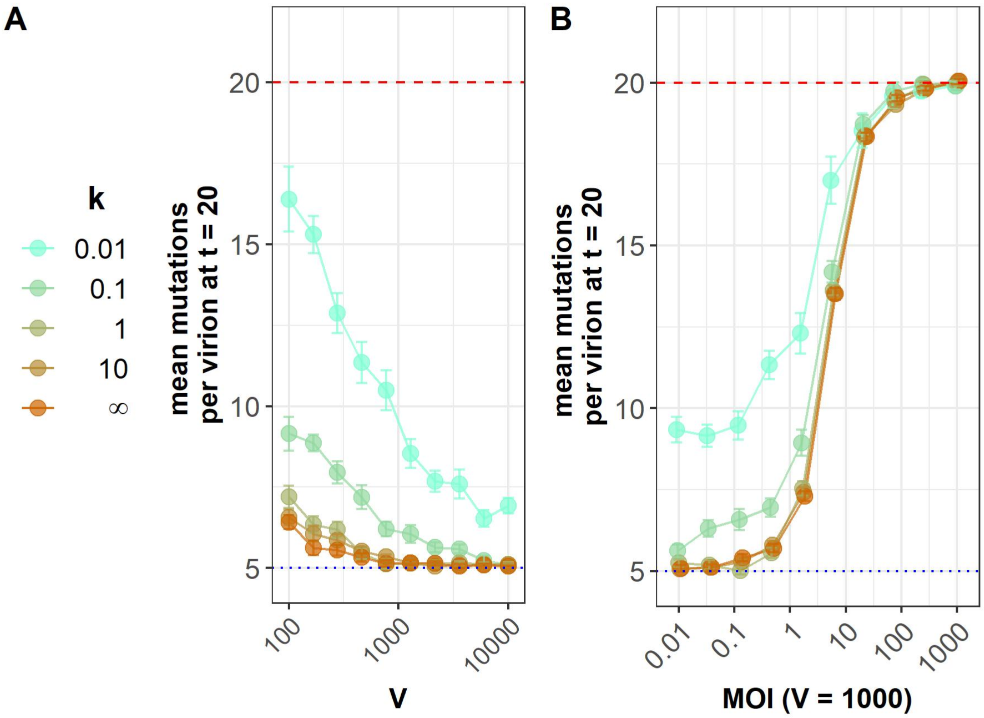

Figure S2: Simulated patterns of stochastic heterogeneity show increases deleterious mutation accumulation with increased heterogeneity. Stochastic heterogeneity is parameterized by k, with $k \ll 1$ corresponding to strong heterogeneity and $k \to \infty$ corresponding to the base model without heterogeneity. All panels show mean number of deleterious mutations after $t = 20$ generations. Each data point shown is the average of 20 replicate simulations with error bars showing the standard error. Red dashed lines show the theoretical expected mutation accumulation at selective neutrality (Ut). Blue dotted lines show the average number of mutations for an infinite viral population size at its mutation-selection balance (U/s). Parameters are $U = 1, s = 0.2, y = 1$, with values of k indicated by color. **(A)** At a constant MOI of 0.1, stochastic heterogeneity increases mutation accumulation. Viral and cell population sizes are increased to maintain the same MOI across simulations. **(B)** In these simulations, the virus population size is kept constant at $V = 1000$ and cell population sizes are modified to change MOI. Different k in (A) and (B) show that additional stochastic heterogeneity (small k) leads to additional mutation accumulation until phenotypic hiding is prominent.

3 Input-Dependent Heterogeneity

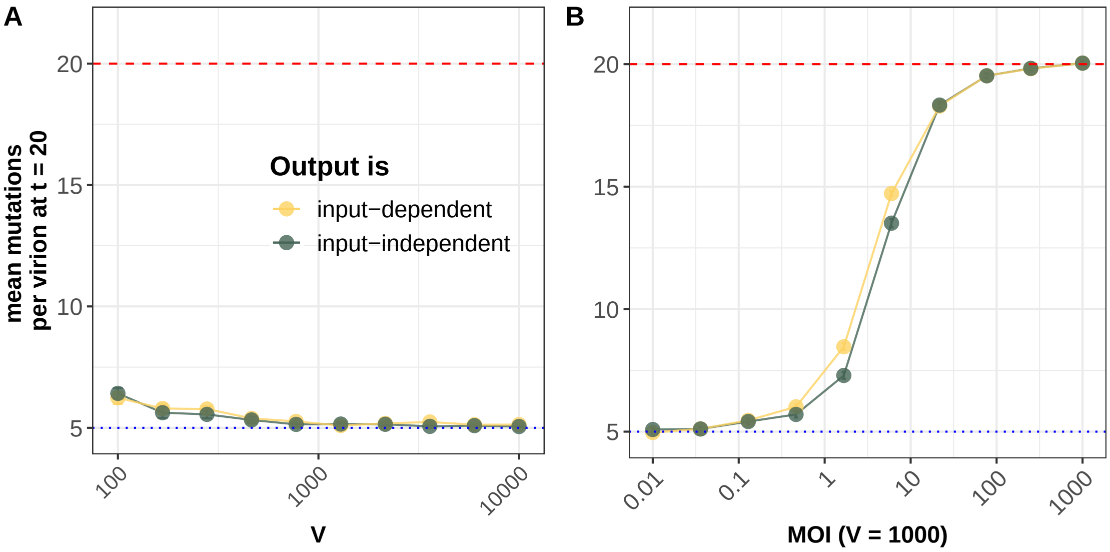

Figure S3: Input-dependent cellular fitness values minimally affect mutation accumulation patterns. The black line represents simulations where there is no input-output relationship (i.e. the base model). The yellow line shows simulations where we have implemented our input-dependent model. Parameters are $U = 1$ and $s = 0.2$. Each data point shown is the average across 20 replicate simulations with error bars showing the standard error. Red dashed lines show the theoretical expectation of mutation accumulation at selective neutrality (Ut). Blue dashed lines show the expectation of mutation accumulation for an infinite viral population size at its mutation-selection balance (U/s). **(A)** Average number of mutations harbored by an individual virion at generation $t = 20$ where MOI is 0.1. In the absence of coinfection, input-output dependence has no impact on deleterious mutation accumulation. **(B)** Average number of mutations harbored by an individual virion at generation $t = 20$. The virus population size is kept constant at $V = 1000$ and cell population sizes are modified to change MOI.

4 Stochastic Heterogeneity Impacts Are Consistent Across Alternative Fitness Functions

4.1 Simulated mutations are assumed to be "dominant"

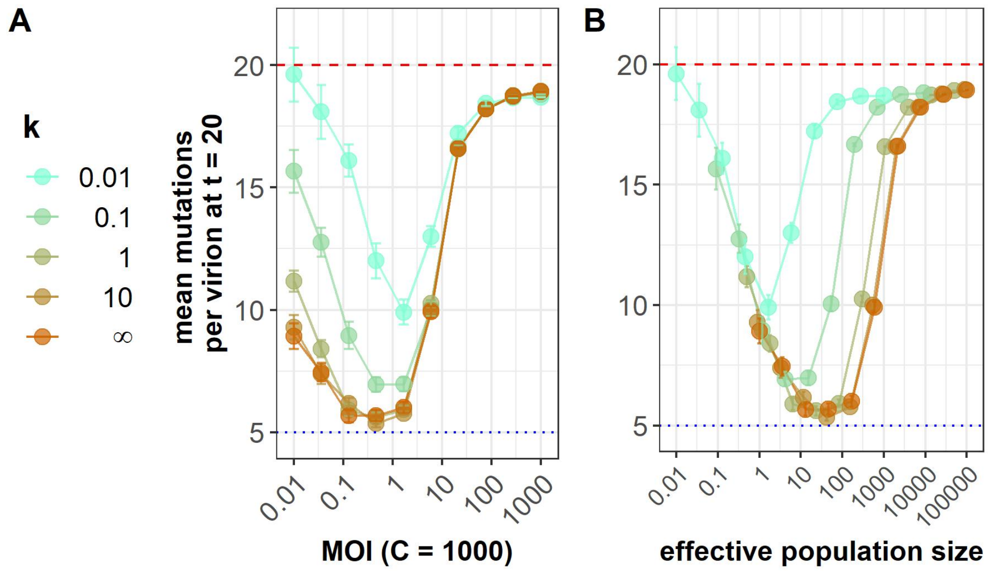

Figure S4: Stochastic heterogeneity increases deleterious mutation accumulation when genic fitness takes the form $\omega_i = \min\{\omega_{i,1}, \ldots, \omega_{i,m}\}$ as described in the Methods of the main text. **(A)** Mean number of deleterious mutations accumulated after $t = 20$ generations across a range of MOI, for different levels of stochastic heterogeneity. Here, the number of cells is kept constant at $C = 1000$, while the number of virions is increased to increase MOI. Stochastic heterogeneity has the largest effect at low MOI. At high MOI phenotypic hiding makes selection ineffective even in the absence of heterogeneity. **(B)** Mean number of deleterious mutations accumulated by $t = 20$ generations across simulations according to their predicted effective viral population size $V_e = V/(1 + 1/k)$. The collapse of the different curves on the left side of the plot shows that V_e accurately captures the effect of heterogeneity on mutation accumulation in the regime where stochasticity is strong − small populations. In both panels, each data point shown is the average of 20 replicate simulations with error bars showing the standard error (with the exception of the three largest population sizes in which show only a single simulation). Red dashed lines show the theoretical expected mutation accumulation at selective neutrality (Ut). Blue dotted lines show the average number of mutations for an infinite viral population size at its mutation-selection balance (U/s). Parameters are $C = 1000, U = 1, s = 0.2, y = 1$.

4.2 Simulated mutations are assumed to be "recessive"

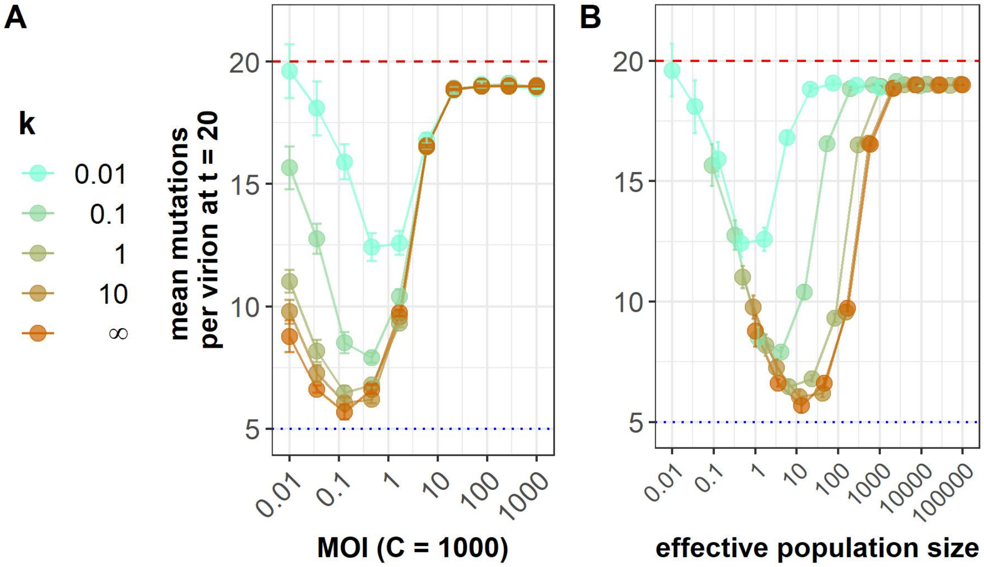

Figure S5: Stochastic heterogeneity increases deleterious mutation accumulation when genic fitness takes the form $\omega_i = \max\{\omega_{i,1}, \ldots, \omega_{i,m}\}$ as described in the Methods of the main text. **(A)** Mean number of deleterious mutations accumulated after $t = 20$ generations across a range of MOI, for different levels of stochastic heterogeneity. Here, the number of cells is kept constant at $C = 1000$, while the number of virions is increased to increase MOI. Stochastic heterogeneity has the largest effect at low MOI. At high MOI phenotypic hiding makes selection ineffective even in the absence of heterogeneity. **(B)** Mean number of deleterious mutations accumulated by $t = 20$ generations across simulations according to their predicted effective viral population size $V_e = V/(1 + 1/k)$. The collapse of the different curves on the left side of the plot shows that V_e accurately captures the effect of heterogeneity on mutation accumulation in the regime where stochasticity is strong − small populations. In both panels, each data point shown is the average of 20 replicate simulations with error bars showing the standard error (with the exception of the three largest population sizes in which show only a single simulation). Red dashed lines show the theoretical expected mutation accumulation at selective neutrality (Ut). Blue dotted lines show the average number of mutations for an infinite viral population size at its mutation-selection balance (U/s). Parameters are $C = 1000, U = 1, s = 0.2, y = 1$.

References

I. Gordo and B. Charlesworth. The degeneration of asexual haploid populations and the speed of Muller's ratchet. *Genetics*, 154(3):1379–1387, 2000.
